# Mapping of the supplementary motor area using repetitive navigated transcranial magnetic stimulation

**DOI:** 10.3389/fnins.2023.1255209

**Published:** 2023-10-04

**Authors:** Giulia Kern, Miriam Kempter, Thomas Picht, Melina Engelhardt

**Affiliations:** ^1^Department of Neurosurgery, Charité - Universitätsmedizin, Corporate Member of Freie Universität Berlin and Humboldt-Universität zu Berlin, Berlin, Germany; ^2^Einstein Center for Neurosciences, Charité – Universitätsmedizin, Corporate Member of Freie Universität Berlin and Humboldt-Universität zu Berlin, Berlin, Germany; ^3^Cluster of Excellence Matters of Activity, Image Space Material, Humboldt-Universität zu Berlin, Berlin, Germany; ^4^International Graduate Program Medical Neurosciences, Charité – Universitätsmedizin, Corporate Member of Freie Universität Berlin and Humboldt-Universität zu Berlin, Berlin, Germany

**Keywords:** supplementary motor area, TMS, brain mapping, motor function, preoperative diagnostic

## Abstract

**Background:**

The supplementary motor area (SMA) is important for motor and language function. Damage to the SMA may harm these functions, yet tools for a preoperative assessment of the area are still sparse.

**Objective:**

The aim of this study was to validate a mapping protocol using repetitive navigated transcranial magnetic stimulation (rnTMS) and extend this protocol for both hemispheres and lower extremities.

**Methods:**

To this purpose, the SMA of both hemispheres were mapped based on a finger tapping task for 30 healthy subjects (35.97 ± 15.11, range 21–67 years; 14 females) using rnTMS at 20 Hz (120% resting motor threshold (RMT)) while controlling for primary motor cortex activation. Points with induced errors were marked on the corresponding MRI. Next, on the identified SMA hotspot a bimanual finger tapping task and the Nine-Hole Peg Test (NHPT) were performed. Further, the lower extremity was mapped at 20 Hz (140%RMT) using a toe tapping task.

**Results:**

Mean finger tapping scores decreased significantly during stimulation (25.70taps) compared to baseline (30.48; *p* < 0.01). Bimanual finger tapping led to a significant increase in taps during stimulation (28.43taps) compared to unimanual tapping (*p* < 0.01). Compared to baseline, completion time for the NHPT increased significantly during stimulation (baseline: 13.6 s, stimulation: 16.4 s; *p* < 0.01). No differences between hemispheres were observed.

**Conclusion:**

The current study validated and extended a rnTMS based protocol for the mapping of the SMA regarding motor function of upper and lower extremity. This protocol could be beneficial to better understand functional SMA organisation and improve preoperative planning in patients with SMA lesions.

## 1. Introduction

The involvement of the supplementary motor area (SMA) in motor and language function has made this cortical area an interest of current research. Damage to this region due to lesion growth or surgical procedures can lead to a characteristic combination of symptoms called the SMA syndrome. This involves various degrees of contralateral akinesia and mutism (Laplane et al., 1977; Zentner et al., 1996; Nachev et al., 2008; Pinson et al., 2022). Depending on the location of the lesion, a characteristic pattern of facial, upper limb or lower limb motor impairment is more likely to occur. This anterior to posterior shift in the type of deficit suggests a somatotopic organisation of the SMA, thus highlighting the necessity for a holistic functional assessment. In addition, language deficits seem to only evolve specifically when the anterior part of the left hemispheric SMA is affected (Bannur and Rajshekhar, 2000; Fontaine et al., 2002; Zeharia et al., 2012). Examinations regarding the importance of the hemispheric dominance in motor function are lacking. Although the SMA syndrome is known to occur mostly temporarily, time of recovery differs between days to months. However, in some patients even persisting long-term deficits of fine motor function have been observed (Zentner et al., 1996; Krainik et al., 2004). The mechanisms of recovery are not yet fully understood. A common hypothesis proposes an increased interhemispheric connectivity especially towards the healthy SMA as underlying process (Krainik et al., 2004; Vassal et al., 2017; Oda et al., 2018; Tuncer et al., 2022).

The SMA is located within Brodmann area 6 in the superior frontal gyrus, however it is not segregated by strict anatomical boundaries (Nachev et al., 2008). So far research concerning preoperative risk assessment and exact determination of the SMA location to improve surgical planning is very limited. While most studies have focused on fMRI to map SMA function in the cortex, these results are too spatially unspecific for a detailed preoperative planning (Kokkonen et al., 2009; Wongsripuemtet et al., 2018). Recently, navigated transcranial magnetic stimulation (nTMS) over the SMA has been found effective to induce errors in executing fine motor skills using the upper extremity (Schramm et al., 2019, 2020). Furthermore, a protocol for mapping of the SMA with a higher spatial resolution compared to fMRI using repetitive nTMS (rnTMS) has been proposed. This protocol used a finger tapping task to localise upper extremity motor function in the SMA of the dominant hemisphere in healthy subjects (Engelhardt et al., 2023).

The aim of this study was to validate and extend the suggested protocol, while focusing on the involvement of the SMA in motor function especially. Specifically, both hemispheres were measured and a protocol extension for the mapping of the lower extremity has been developed. In the long run, this could be used to acquire a better understanding of the functional organisation of the SMA and to establish a non-invasive SMA mapping protocol within the clinical setting to improve risk assessment and preoperative diagnostics.

## 2. Methods

### 2.1. Ethics

This study was approved by the Ethics Committee of the Charité Universitätsmedizin Berlin and conducted in accordance with the Declaration of Helsinki. Written informed consent was provided by each participant.

### 2.2. Participants

30 healthy subjects (35.97 ± 15.11, range 21–67 years; 14 females) above the age of 18 were recruited for this prospective study. They all had no history of neurological or psychological diseases and met the criteria for receiving an nTMS and MRI. This includes no history of epilepsy or seizures also within the family, migraine, tinnitus, pregnancy, metallic implants (e.g., pacemaker, cochlear implants, intrauterine devices), intake of prescription drugs within the past 14 days and permanent makeup. One additional subject (55 years, female) was excluded from the study due to a high RMT (resting motor threshold) which precluded that required stimulation intensities could be reached.

### 2.3. MRI

Each participant received a T1-weighted structural MRI (MPRAGE, TR = 2.530 ms, TE = 4.94 ms, TI = 1.100 ms, flip angle = 7, voxel size = 1 mm × 1 mm × 1 mm, 176 slices) measured on a Siemens 3-T Magnetom Trio MRI scanner (Siemens AG, Erlangen, Germany) as individual navigational data for the nTMS.

### 2.4. Neuronavigated TMS

Using the navigated brain stimulation system (NBS 5, Nexstim, Helsinki, Finland) with a biphasic figure-of-eight coil (outer diameter: 70 mm) each subject underwent a nTMS session divided into two major components. For each hemisphere, assessment of the primary motor cortex was followed by the SMA mapping always examining the contralateral limb. The starting hemisphere was alternated between participants to avoid confounding of any hemispheric differences due to effects of stimulation order.

### 2.5. Motor mapping

The primary motor cortex was assessed using single pulse nTMS. To examine muscle activity, surface electrodes (Neuroline 720; Ambu, Ballerup, Denmark) connected to the systems’s integrated EMG were attached to the first dorsal interosseus muscle of the corresponding hand. The ground electrode was placed on the left palmar wrist. To keep the muscle output below the threshold of 10 μV all participants were instructed to relax their hand. Subsequently the M1 hotspot was determined as the location, rotation and tilt where reliably the highest muscle responses could be evoked. Afterwards the RMT was assessed using the system’s integrated algorithm (Engelhardt et al., 2019). Furthermore, cortical representation of the target muscle was assessed at 105% of the RMT (Engelhardt and Picht, 2020). This area mapping was performed to delineate motor areas from consequently determined SMA areas.

### 2.6. SMA mapping

Starting with the upper extremity the SMA was mapped using repetitive nTMS (20 Hz, 120% RMT, 5 s bursts, ITI 5 s) with the stimulation coil positioned perpendicular to the interhemispheric cleft (Engelhardt et al., 2023). Subjects were instructed to perform a finger tapping task for 5 s by tapping the index finger as fast as possible (Hiroshima et al., 2014; Schramm et al., 2019; Engelhardt et al., 2023). The number of taps was recorded by the Apple iPad App Counter +. Firstly, a baseline tapping score was acquired as an average of two rounds without stimulation. If a considerable increase in taps occurred over time due to practice effects the baseline was renewed at a later timepoint within the same session. Secondly, the same task was conducted with stimulation for 15 to 21 stimulation points depending on the individual anatomy. The covered SMA area was estimated as posterior part of the superior frontal gyrus rostral to M1 up to the cortical crossing point of a perpendicular line through the anterior commissure (Vorobiev et al., 1998). Subjects started finger tapping with the onset of SMA stimulation. To avoid muscle fatigue, the participants rested their hand for a few minutes after a maximum of seven stimulations. After covering the suspected SMA area, stimulation of each point was repeated in the same order. Afterwards a SMA hotspot was determined as stimulation point with the largest errors and hence the least amount of finger taps on average. To this purpose the two or three stimulation points with the least taps were stimulated again to decide on the final hotspot with the lowest tapping score as an average of three rounds. Further, only points that were unlikely to activate M1 based on RMT and proximity to M1, were considered as SMA hotspot (Table 1).

**Table 1:** SMA mapping results of the upper extremity for 30 healthy subjects with a median of 19 (IQR 18–20) unique stimulation points per hemisphere.

Category	Number of subjects with errors (% of total sample)	Number of stimulation points with errors Median (IQR)	Error incidence in % Median (IQR)
Replicable major errors	11 (37%)	2 (1–3)	10.88 (5.34–15.79)
Replicable minor errors	23 (77%)	1 (1–2)	5.88 (5.13–10.53)
Limited replicable errors	13 (43%)	1 (1–1)	5.26 (5.00–5.72)

Number of stimulation points with errors and error incidences for different subgroups depending on the different error categories: major errors (≥15%), minor errors (10–20%), replicable (same error category in two stimulation rounds), limited replicable (different error category in two stimulation rounds). Median and IQR were calculated for the corresponding subgroups. Subjects are not unique to one category.

For this hotspot, the participants performed a bimanual finger tapping task to investigate bimanual coordination as part of the SMA function. This included tapping with the index fingers of both hands in parallel. This task was repeated three times. Taps of the stimulated hand were recorded to quantify a facilitation of tapping performance (reduction of the reduced error) compared to unimanual tapping. Further, subjects performed a shortened version of the Nine-Hole-Peg Test (NHPT), where they only had to insert pegs into the pegboard to examine the role of the SMA in dexterity. A shortened version was chosen to ensure task completion was feasible within the maximum possible stimulation duration. The time to insert all pegs was recorded for analysis. After two rounds as baseline, stimulation was applied three times for a maximum of 20 s to cover the full task performance.

Next, the lower extremity was mapped using repetitive nTMS (20 Hz, 140% RMT, 10s bursts, ITI 10s) while the subjects performed a toe tapping task. Two rounds of baseline were followed by stimulating 5 to 10 points in the posterior part of the SMA. This region was chosen according to the proposed somatotopy of the SMA (Bannur and Rajshekhar, 2000; Fontaine et al., 2002; Zeharia et al., 2012). Again, each point was stimulated twice. For analysis visually detected movement disruptions in tapping performance were recorded.

### 2.7. Data analysis

All sessions were recorded on video using the nTMS system’s inbuilt camera. For each SMA stimulation point of the upper extremity the induced electric field at the M1 hotspot was compared with the RMT. This was achieved by placing the Nexstim software integrated crosshair on the M1 hotspot during SMA stimulation. The system is then automatically able to show the induced electric field in V/m for both the point of stimulation and the crosshair. If the RMT value was exceeded, the SMA stimulation point was excluded from further analysis. For the remaining points, errors were classified into three categories indicating the reduction in finger taps compared to baseline. A reduction of <10% accounted for no error, 10–20% for minor error and ≥ 20% for major error. A fourth category was used to mark M1 affected stimulation points.

For the lower extremity, potential functional SMA points were stimulated again at rest while EMG activity of the abductor hallucis brevis muscle was recorded. In case of strong muscle responses, this stimulation point was excluded from analysis. Errors were categorised into two groups depending on occurrence or absence of visually detected movement effects compared to baseline by two independent observers. Again, an additional category was used to mark M1 affected stimulation points.

Subsequently error classifications were imported into the NBS software to attain coloured SMA maps on the individual MRIs.

### 2.8. Statistical analysis

The median number of errors and error incidence with their respective interquartile range were calculated for the separate error categories to examine task disruption during stimulation. The focus was on replicable errors only, defined as points with a similar tapping score reduction according to the defined error categories in at least 2 stimulation rounds. In contrast, stimulation points with a tapping score reduction of ≥10% in at least 2 stimulation rounds but within different error categories were defined as limited replicable errors. Furthermore, the effect of SMA stimulation during unimanual and bimanual finger tapping to baseline finger tapping was compared using linear mixed models. Similarly, the impact of SMA stimulation on NHPT performance was assessed. To investigate the impact of hemispheric dominance 3 ambidextrous subjects were excluded leaving a population size of n = 27. Handedness was determined using Edinburgh Handedness Inventory (Oldfield, 1971). The importance of hemispheric dominance on incidence of finger and toe tapping errors during SMA stimulation was evaluated using two-sided Wilcoxon signed-rank test. Specifically, median finger and toe tapping error incidence and interquartile range for each error category was compared between both hemispheres. The level of statistical significance was set to *p* < 0.05. All analyses were performed using R Studio (version 2022.07.2 + 576) with the packages dplyr (Wickham et al., 2023a), car (Fox and Weisberg, 2019), ggplot2 (Wickham, 2016), reshape (Wickham, 2007), tidyverse (Wickham et al., 2019), MASS (Venables and Ripley, 2002), nlme (Pinheiro et al., 2023) and svglite (Wickham et al., 2023b).

## 3. Results

### 3.1. Mapping of the upper extremity

**Figure 1:**
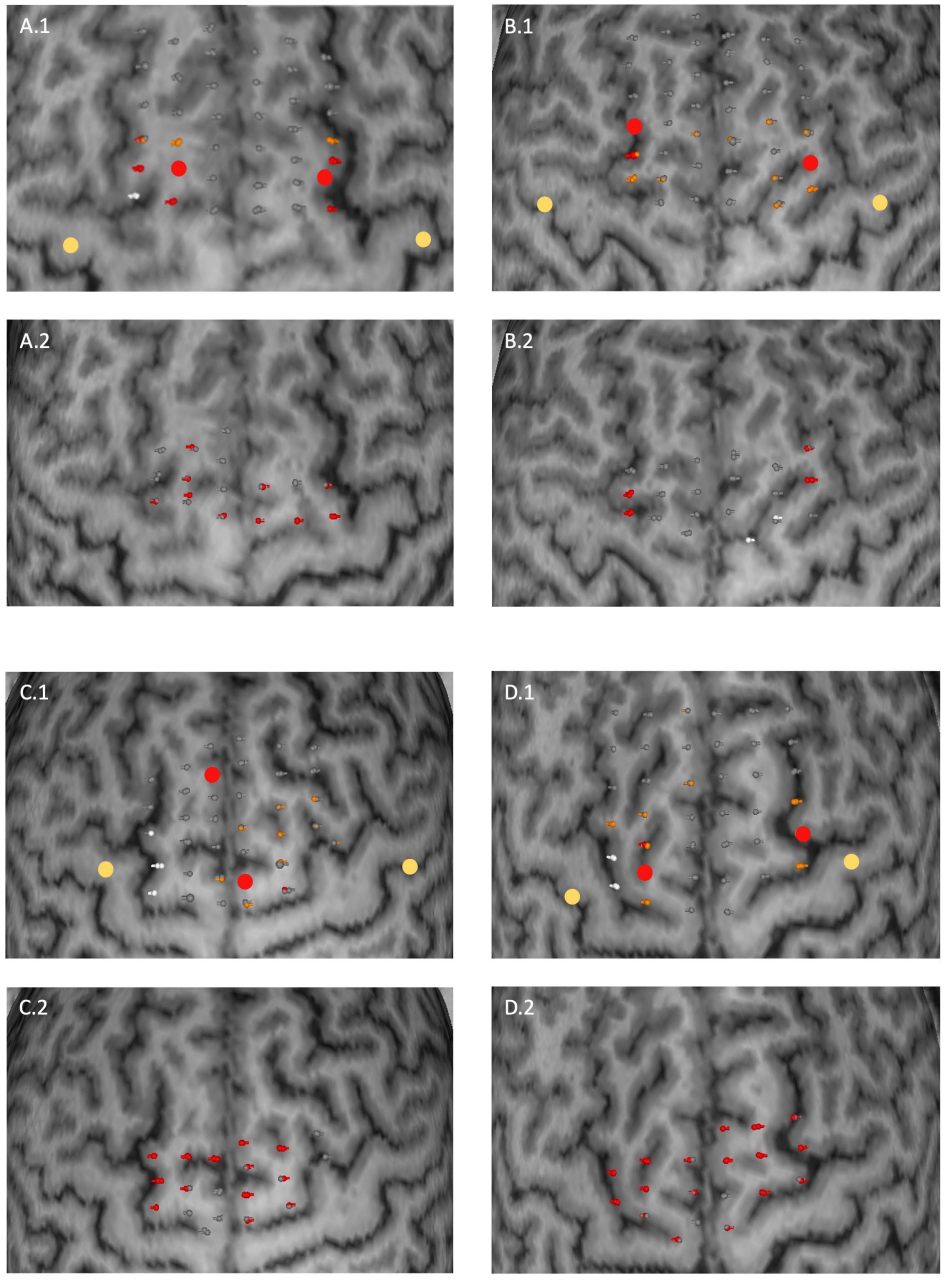
Examples of SMA error maps for four subjects **(A–D)**. Two subplots correspond to one subject for the upper extremity (0.1) and for the lower extremity (0.2). Stimulation points are coloured in their corresponding error category. Upper extremity: grey (no error), orange (minor error), red (major error); lower extremity: grey (no visually detected movement error) and red (visually detected movement error) respectively. White coloured points represent stimulation points for which the residual electrical field at the M1 hotspot field was above the RMT. Larger dots correspond to M1 (yellow) and SMA (red) hotspots. The distance between two stimulation points was 4 to 5 mm on average.

A median of 19 (IQR 18–20) unique points was stimulated across all participants. Replicable errors during the finger tapping task could be induced in 24 out of 30 healthy subjects for at least one hemisphere. Among those, 13 exhibited replicable errors for both hemispheres. In 11 subjects, stimulation led to replicable major errors over a median of 2 (IQR 1–3) points across all hemispheres. Hence, the median error incidence for these subjects was 10.88% (IQR 5.34–15.79%). 23 subjects showed replicable minor errors with a median of 1 (1–2) replicable minor error and a median error incidence of 5.88% (5.13–10.53%) accordingly. Limited replicable errors occurred in 13 subjects over a median of 1 (1–1) stimulation points. The median error incidence for these participants was 5.26% (5.00–5.72%). These results are summarized in Table 1. Overall, there were strong interindividual differences between the occurrence of errors as well as size and distribution of error maps. Some examples of SMA error maps are presented in Figure 1.

### 3.2. Additional tasks

**Figure 2:**
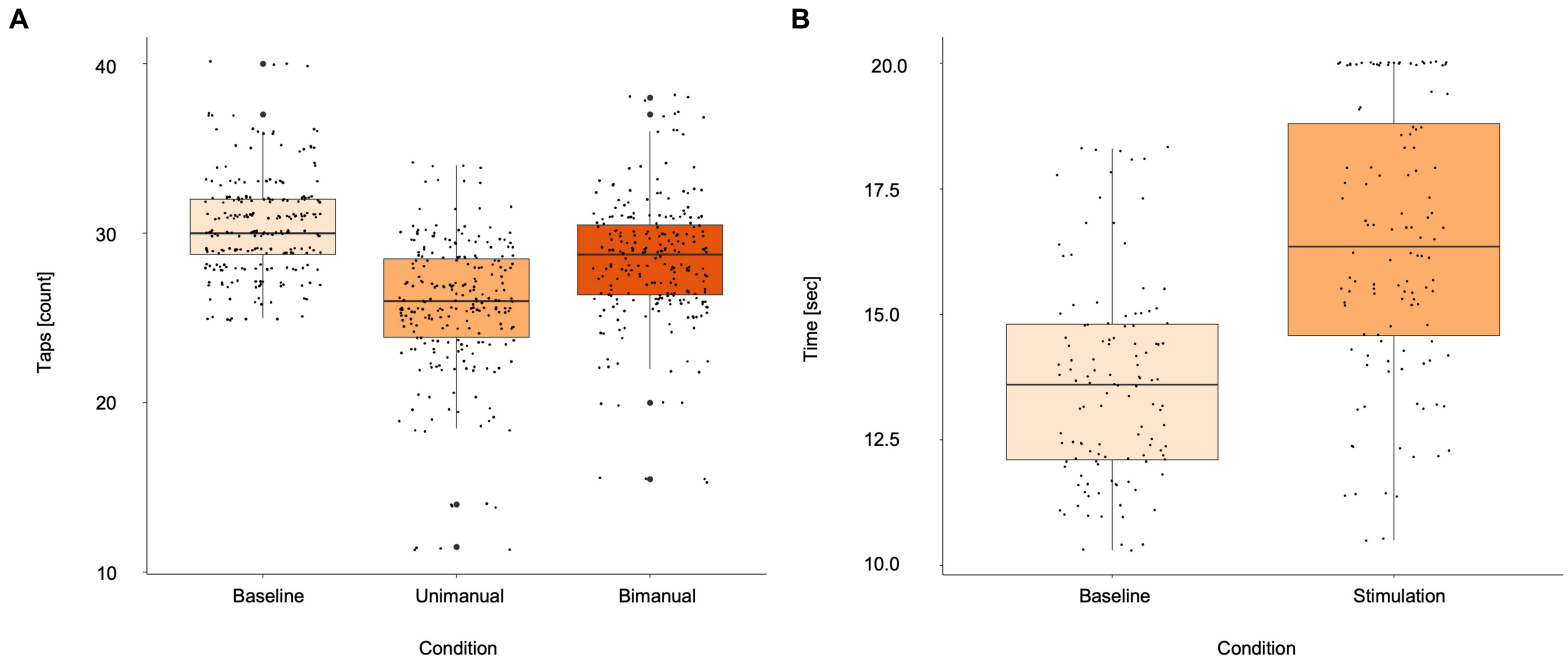
Boxplots for **(A)** finger tapping scores during baseline, unimanual and bimanual tapping during stimulation, **(B)** Nine-Hole Peg Test during baseline and stimulation. Asterisks indicate statistically different effects (*p* < 0.05; linear mixed models). Small black dots represent single subject values.

A significant reduction of finger taps occurred during stimulation (25.70 ± 4.00 taps) compared to baseline (30.48 ± 2.94 taps; *p* < 0.01). This effect was not impacted by subjects’ age (*p* = 0.2604). Bimanual finger tapping increased the number of taps (28.43 ± 3.74 taps) compared to unilateral tapping during stimulation significantly (*p* < 0.01). However, the number of finger taps in the bimanual condition still remained below baseline (*p* < 0.01; Figure 2A). An example of the different finger tapping task conditions for two subjects can be found in Supplementary Videos. Completion time for the NHPT increased significantly during stimulation (16.4 ± 4.2 s) compared to baseline (13.6 ± 2.7 s; *p* < 0.01; Figure 2B) as demonstrated for one subject in Supplementary Videos. In 11 cases (14 hemispheres), the intensity for the NHPT had to be reduced in 5% steps due to system inbuilt safety restrictions forbidding longer stimulation for the necessary completion time. The intensity was 110% for 9 hemispheres and 105% for 3 hemispheres. The lowest applied stimulation intensity was 100% for 2 hemispheres. One subject specifically described a built-up of stimulation effect on the finger tapping task over time.

### 3.3. Mapping of the lower extremity

For the lower extremity, a median of 10 (9–10) unique stimulation points was set per hemisphere across all participants. Replicable visually detected movement errors could be induced in 28 out of 30 subjects (20 bi-hemispherically, 28 uni-hemispherically) for a median of 2 (1–3). Hence, the median error incidence for the lower extremity in these subjects was 20.00% (11.11–33.33%). The exact type of visually detected movement errors varied between subjects. Increased arrhythmicity and reduced fluency in toe tapping was common (Supplementary Videos. Subject 1). In addition, some subjects showed sudden complete arrest of tapping or proceeded with the task even after the stimulation had stopped (Supplementary Videos. Subject 2). For 3 subjects the stimulation intensity was reduced to 130% (2 left-hemispherically, 1 right-hemispherically) due to a very high RMT.

### 3.4. Impact of hemispheric dominance

Data of 22 right-handed and 5 left-handed participants was included in this analysis. A median of 19 unique points was stimulated for both dominant (IQR 18–20) and non-dominant (IQR 19–20) hemisphere for the upper extremity across subjects. For participants with major errors the median error incidence was 11.76% (5.56–16.67%) for the dominant and 10.12% (5.20–15.20%) for the non-dominant hemisphere accordingly (*p* = 0.075). For minor errors the median error incidence was 5.88% (5.00–10.53%) for the dominant and 5.56% (5.26–10.39%) for the non-dominant hemisphere (*p* = 0.672). Subjects with limited replicable errors showed a median error incidence of 5.26% (5.00–5.88%) for the dominant and 5.00% (5.00–5.26%) for the non-dominant hemisphere (*p* = 0.154). For the lower extremity a median of 10 unique points (9–10) was stimulated across participants for the dominant hemisphere. The non-dominant hemisphere received the same amount of unique stimulation points. A median error incidence of 20.00% (20.00–33.33%) was evaluated for the dominant hemisphere across subjects with visually detected movement errors. For the non-dominant hemisphere, it was 18.33% (10.28–30.00%) respectively (*p* = 0.204). Overall, differences in error incidences between the dominant and non-dominant hemisphere did not reach the level of significance.

## 4. Discussion

This study validated a non-invasive rnTMS based protocol for SMA mapping in healthy subjects. The current findings underline the feasibility of an extension of the proposed protocol to the non-dominant hemisphere and lower extremity. Further, refined instructions for mapping procedures and error classifications were provided. Finally, the present study gives insights into the somatotopic organisation of the SMA.

Following the virtual lesion paradigm, the current results are in line with previous studies showing that rnTMS applied to the SMA can induce a reduction of finger taps (Schramm et al., 2019; Engelhardt et al., 2023). Mapping of both hemispheres was possible similarly to preceding studies which used the Jebsen-Taylor hand function test (Schramm et al., 2019, 2020). However, the importance of hemispheric dominance regarding SMA function is not yet fully understood. The mentioned studies report a stronger effect during stimulation of the right hemisphere when looking at right-handed subjects while the current results suggest no significant differences. Other studies linked the occurrence of language deficits to the resection of the dominant hemisphere (Bannur and Rajshekhar, 2000; Dalacorte et al., 2012). In this study, only the SMA involvement in motor function was investigated. Overall, the relevance of hemispheric dominance should be investigated by future studies.

The current study refined and standardised the protocol description and error classification of the previously proposed protocol (Engelhardt et al., 2023). According to this new protocol a minimum of 15 points was stimulated twice per hemisphere and subject. In this context error categories for the finger tapping reduction have been lowered from ≥30% to ≥20% for major errors and ≥ 15% to ≥10% for minor errors. Overall, protocol changes were made specifically with the focus on ensuring replicability of errors.

The previously observed built up of stimulation effects (Engelhardt et al., 2023) was only observed in one subject in the present study. This raises the question whether the effect was indeed related to stimulation or rather a characteristic of subject dependent muscle fatigue. In support of an actual stimulation induced effect, Emanuel et al. (2021) report an increased stuck-in-the-middle phenomenon meaning a sharper decrease in effort towards the middle of a task compared to beginning and end after inhibitory SMA stimulation.

The current results suggest an improvement of finger tapping performance due to bimanual instead of unimanual tapping during stimulation. It has been shown that SMA activation can drive also contralateral executive motor function in case of contralateral SMA failure through transcallosal connections. This is supported by the notion that a strong interhemispheric connectivity facilitates rehabilitation after SMA lesions (Krainik et al., 2004; Vassal et al., 2017). Previous studies have shown the involvement of the SMA in coordinating bimanual movements by altering the interhemispheric connectivity (Serrien et al., 2002; Welniarz et al., 2019). Therefore, a possible explanation might be that bimanual tapping compensates for stimulation induced disruptions. Further, it could be hypothesised that a short delay in tapping normalisation might occur due to the time needed for interhemispheric transmission.

Overall, the present study suggests stimulation effects smaller than reported by Engelhardt et al. (2023). Responsiveness could be increased with higher stimulation intensities while controlling for activation of M1yet at the cost of spatial specificity. The importance of ensuring proper SMA responses by controlling for the residual electric field over M1 was reinforced in the current study. A high interindividual difference regarding size and location of the area susceptible to stimulation was found similarly to the preceding study (Engelhardt et al., 2023). This could be partially explained by functio-anatomical differences or variable effects of methodology. Therefore, we suggest caution when interpreting the absence of errors as this could be due to the absence of function or due to the lack of a sufficiently intense simulation. In contrast, induced errors could be more reliable. However, these hypotheses need to be investigated in a clinical sample, where the relation between presence or absence of errors, a potential resection and postoperative deficits can be clearly established.

Previous studies highlight an underlying somatotopy of the SMA mostly based on the clinical outcome after SMA resection. These findings indicate a structural organisation of face, upper and lower extremity from anterior to posterior (Zentner et al., 1996; Fontaine et al., 2002; Krainik et al., 2004). The current results support this hypothesis as errors in upper extremity function occurred mostly in the medial part of the SMA and errors in lower extremity function in the posterior part. However, due to time constraints of the measurement not all SMA portions were examined for lower extremity errors, thus limiting these conclusions. Nevertheless, the present study demonstrates that a somatotopic map could be created using more tasks and testing upper and lower extremities.

As a next step, this refined protocol could be applied to patients to validate whether rnTMS positive stimulation points are functionally essential and therefore rnTMS based SMA mapping could deliver valuable clinical information. In this context, the protocol could be implemented within the clinical setting to aid risk assessment in addition to preoperative diagnostics and planning. Further, it could be used to assess SMA reorganisation due to surgery or other brain lesions by comparing SMA maps of different timepoints.

### 4.1. Limitations

The present study focused on the number of finger taps as a simple and easy to assess outcome. The toe tapping has been analysed regarding visually detected movement errors by two independent assessors. Future studies could use more detailed and objective measures by applying a sensor to measure timing of taps, inter-tap intervals or movement kinematics. In addition, these measures could be used to investigate a potential built-up of the SMA stimulation effect over time. Further, these analyses could aid to identify mechanisms behind bimanual movement coordination including whether the contralesional SMA takes over function of the lesioned side. Electric field estimates were based on the multi spherical head model integrated in the Nexstim system to enable fast and easy online assessment. However, more realistic head models might lead to slightly deviating results of the electric field estimates (Nieminen et al., 2022). These differences might become relevant when stimulating close to M1 or with residual intensities close to the RMT. Especially for the lower extremity activation of the contralateral SMA cannot be completely excluded due to the high stimulation intensities used and proximity of both areas. Even though the strongest stimulation effects were observed a bit more distant from the midline, a potential confounding activation of the contralateral SMA should be carefully monitored. The current study focused on anatomical landmarks to identify the stimulation area, however SMA boundaries are not strictly defined (Nachev et al., 2008). Sites inducing foot movement disruptions also encompassed sites which produced major disruptions in finger tapping. This suggests that given the existence of a somatotopy, boundaries between hand and foot areas might not be sharp. Future studies could stimulate more frontal or lateral regions such as the pre-SMA to further investigate spatial delineation and somatotopic organisation of the SMA. This could also aid to additionally validate SMA specificity of stimulation effects.

### 4.2. Conclusion

The present study refined and validated a protocol for the non-invasive rnTMS based mapping of the SMA considering both hemispheres and somatotopy of the SMA. As a next step, this protocol will be tested in a clinical setting to test its ability to aid preoperative diagnostics, risk assessment for the occurrence of the SMA syndrome and assessment of postoperative reorganisation in brain tumor patients.

## Data availability statement

The original contributions presented in the study are included in the article/Supplementary Material, further inquiries can be directed to the corresponding author.

## Ethics statement

The studies involving humans were approved by Ethics Committee of the Charité Universitätsmedizin Berlin. The studies were conducted in accordance with the local legislation and institutional requirements. The participants provided their written informed consent to participate in this study.

## Author contributions

GK: Conceptualization, Data curation, Formal analysis, Investigation, Methodology, Project administration, Visualization, Writing – original draft. MK: Conceptualization, Methodology, Writing – review & editing. TP: Conceptualization, Methodology, Supervision, Writing – review & editing. ME: Conceptualization, Data curation, Formal analysis, Methodology, Project administration, Supervision, Writing – review & editing.
