# Supplementary material for: Mapping of the supplementary motor area using repetitive navigated transcranial magnetic stimulation
Source: Front Neurosci. 2023 Oct 4;17:1255209. doi: 10.3389/fnins.2023.1255209 (PMC10582562; doi:10.3389/fnins.2023.1255209)
Supplement: Supplementary file 1 [file Table_1.PDF]

Category	Number of subjects with errors (% of total sample)	Number of stimulation points with errors Median (IQR)	Error incidence in % Median (IQR)
**Replicable major errors**	11 (37%)	2 (1-3)	10.88 (5.34-15.79)
**Replicable minor errors**	23 (77%)	1 (1-2)	5.88 (5.13-10.53)
**Limited replicable errors**	13 (43%)	1 (1-1)	5.26 (5.00-5.72)
